# A Legal Outrage

**Published:** 1892-01

**Authors:** 


### A LEGAL OUTRAGE.

LAWS are usually passed for the protection of communities and individuals, and in order that equal justice may be administered, courts have been established. This, theoretically, is the basis of all law; but, unfortunately, this beneficent idea often fails through various causes, not the least of which may be found in the officious intermeddling of individuals and societies. This truism has been made clearly apparent in the effort to enforce the dental enactments of the various States. In some, societies have been modelled after the "Law and Order Associations." The motive for the origin of these has, doubtless, been of the most unselfish character, but history is full of examples of such bodies finally becoming odious from their tyrannical proceedings.

Our attention was recently called to a case occurring in Philadelphia which illustrates this view. There a society has been organized the sole object of which is the prosecution of violators of the dental law of Pennsylvania. Now, this law is one of the most satisfactory in the statute books of the various States, and if left to its natural development it would prove of greater value in the future than it has in the past, valuable as that has been.

The progressive steps taken by the organization alluded to has finally led, very naturally, to overstepping the bounds laid out for its government, and, if continued, will certainly bring the whole question of dental law into contempt.

The law requires, very properly, that all practitioners should be registered; but there has been a good deal of misunderstanding in regard to this, and many mistakes have been made by individuals without any intention of violating any of its provisions. This is especially true of recent graduates.

The special case that calls for notice is that of a young practitioner of Philadelphia. This gentleman, by his scientific work, has made for himself an enviable reputation both at home and abroad. Possibly for this reason he was selected as a shining mark for this ill-advised action. Without previous notice, a warrant was issued, and the officer of the law waited on him while engaged in his professional duties. The officer insisted on serving the warrant at once, and it required argument and persuasion to prevent him from dragging the dentist through the streets of the city as a common criminal. Indeed, had this minion of the law performed his duty, such would have been the result. The matter will be settled in the courts, and we can there safely leave it.

What, however, is to be thought of men who openly violate in this manner, under the guise of a public duty, all professional practice? It seems to us that the societies to which they may belong have a duty to perform, and if they have any proper self-respect they will promptly place such individuals beyond the pale of professional recognition. They can have nothing in common with those who appreciate professional ethics or gentlemanly procedures.

